# Temporal changes of haematological and radiological findings of the COVID-19 infection—a review of literature

**DOI:** 10.1186/s12890-020-01389-z

**Published:** 2021-01-22

**Authors:** Albert Yick Hou Lim, Jun Leng Goh, Mae Chui Wei Chua, Bee Hoon Heng, John Arputham Abisheganaden, Pradeep Paul George

**Affiliations:** 1 Department of Respiratory and Critical Care Medicine, Tan Tock Seng Hospital, National Healthcare Group, 11 Jalan Tan Tock Seng, Singapore, 308433 Singapore; 2 Yong Loo Lin School of Medicine, National University of Singapore, Singapore, Singapore; 3 Lee Kong Chian School of Medicine, Nanyang Technological University, Singapore, Singapore; 4 Health Services and Outcomes Research, National Healthcare Group, Singapore, Singapore; 5 Faculty of Health Sciences, University of Adelaide, Adelaide, Australia

**Keywords:** COVID-19, SARS-COV-2, Temporal trends, Clinical manifestations, Lymphocyte count, Lymphopenia, Neutrophil count, C-reactive protein, Lactate dehydrogenase, CT thorax imaging, Pneumonia

## Abstract

**Background:**

COVID-19 is a systemic viral infection which mainly targets the human respiratory system with many secondary clinical manifestations especially affecting the hematopoietic system and haemostasis. Few studies have highlighted the prognostic value of blood findings such as lymphopenia, neutrophil/lymphocyte ratio, platelet/lymphocyte ratio, LDH, CRP, cardiac troponin, low-density lipoproteins and chest radiographic abnormality. A study of progressions of blood and radiological results may help to identify patients at high risk of severe outcomes. This systematic review aimed to assess the temporal progression of blood and radiology findings of patients with COVID-19.

**Methods:**

Comprehensive systematic literature search was conducted on Medline, Embase and Cochrane databases to identify articles published for peripheral blood investigation and radiological results of COVID-19 patients.

**Results:**

A total of 27 studies were included in this review. The common laboratory features reported include lymphopenia, elevated levels of C-reactive proteins and lactate dehydrogenase. For radiological signs, ground-glass opacifications, consolidations, and crazy paving patterns were frequently reported. There is a correlation between lymphocyte count, neutrophil count and biomarkers such as C-reactive proteins and lactate dehydrogenase; at a later phase of the disease (more than 7 days since onset of symptoms), lymphopenia worsens while neutrophil count, C-reactive protein levels and lactate dehydrogenase levels increase. Frequencies of ground-glass opacifications and ground-glass opacifications with consolidations decrease at a later phase of the disease while that of consolidation and crazy paving pattern rises as the disease progresses. More extensive lung involvement was also seen more frequently in the later phases.

**Conclusion:**

The correlation between temporal progression and the reported blood and radiological results may be helpful to monitor and evaluate disease progression and severity.

## Background

In early December 2019, a series of pneumonia of unknown causes with clinical features that resemble viral pneumonia were identified in Wuhan, Hubei, China [1]. The World Health Organisation (WHO) officially named the clinical condition COVID-19 (coronavirus disease-19) [2] and the Coronavirus Study Group of the International Committee on Taxonomy of Viruses renamed the virus “severe acute respiratory syndrome coronavirus 2” (SARS-CoV-2) [3]. Coronaviruses belong to the family *Coronaviridae* and the order *Nidovarales*, a family that includes viruses that cause diseases ranging from the common cold to severe acute respiratory syndrome (SARS) and the Middle East respiratory syndrome (MERS) [4]. WHO has since characterised COVID-19 as a global pandemic [5]. The WHO Coronavirus Disease (COVID-19) Dashboard reflects that 220 countries have been affected, with 58,425,681 confirmed cases and 1,385,218 deaths globally as of 23 November 2020.

The incubation period for COVID-19 was estimated to be 5.1 days, and 97.5% became symptomatic within 11.5 days [6]. The main clinical symptoms include fever, cough, fatigue, sputum production, shortness of breath and myalgia/arthralgia [1]. Other minor symptoms include headache or dizziness, diarrhoea, nausea and vomiting [7]. Major complications of patients with COVID-19 include acute respiratory distress syndrome (ARDS) and some also progress to multi-organ failure [8]. The severity of patients with COVID-19 runs on a spectrum from mild, severe to critical [9].

Given the wide spectrum of severity that can be found in a patient with COVID-19, it is important to identify potential clinical characteristics that would help predict the clinical outcome early. This in turn could guide management in terms of resource allocation. Thus far, several potential predictors of outcome have been suggested to help monitor patients which may develop severe complications. Lymphopenia has been shown to be a prominent laboratory finding in severe COVID-19 patients and is important in predicting the prognosis [10, 11]. Liu et al. [12] has also reported that neutrophil-to-lymphocyte ratio is an independent risk factor for in-hospital mortality of patients. Other potential haematological predictors of outcome that have been proposed included C-reactive proteins (CRP) levels [13], lactate dehydrogenase (LDH) levels [14], cardiac troponin-I [15], low-density lipoproteins [16]. In a recently published study, Liang et al. [17] proposed a new clinical risk score to predict the occurrence of critical illness in hospitalised COVID-19 patients. The risk score included many of the above-mentioned laboratory markers as well as chest radiographic abnormality. It may also be fruitful to look at the temporal progression of blood and radiological results whilst comparing between patient groups with a mild disease course and those with a severe outcome or even death. Insights drawn can demonstrate typical progressions of blood and radiological results which may help to identify patients at high risk of severe outcomes.

It should also be recognised that a significant proportion of infected patients are asymptomatic in the early days after documented exposure, with an estimated proportion to be about 18% [18]. In fact, higher proportions of asymptomatic cases were reported in some studies with 87.8% of the patients who were tested positive being asymptomatic [19]. The concern lies in the fact that though asymptomatic, these carriers are still very capable of transmitting COVID-19 via person-to-person contact, thereby propagating the pandemic [20]. Therefore, being able to identify such patients at an early stage via haematological or radiological investigations would be beneficial, especially in regions with limited availability of and accessibility to COVID-19 test kits.

## Methods

A systematic literature review was conducted using the online databases, Pubmed, Embase and Cochrane databases. The search was done with the aim of identifying publications that reported the radiological or blood changes in patients with COVID-19.

This was done in accordance with the Cochrane Handbook of Systematic Reviews and Meta-analysis and Preferred Reporting Items for Systematic Reviews and Meta-Analyses (PRISMA) statement guidelines [21]. The electronic searches covered the period from 1 December 2019 to 29th March 2020. They were done using a combination of Medical Subject Headings (MeSH) and non-MeSH key terms with Boolean operators (Additional files 1 to 6).

All searches underwent double-blind screening of the title and abstract by two researchers for inclusion and exclusion criteria. The inclusion criteria are studies that include patients diagnosed with COVID-19 and report radiological (computed tomography (CT) of thorax/chest radiograph) or blood (full blood count) findings together with their date since onset of symptoms when the respective investigations were done. Case reports, studies with fewer than 10 patients, abstracts, letters to editors, editorials, commentaries, features, news, guidelines, opinion pieces and non-English language papers were excluded. Duplicates were removed, and the selected articles underwent a full-text review to verify quality and eligibility. Conflicts were resolved by consensus.

Data was extracted from text, tables and figures in each selected paper using a standardised method into a preformatted database by a single researcher. All extracted data was then verified by a second researcher independently. A sample of the extraction grid is included in Additional file 2.

**Fig. 1:**
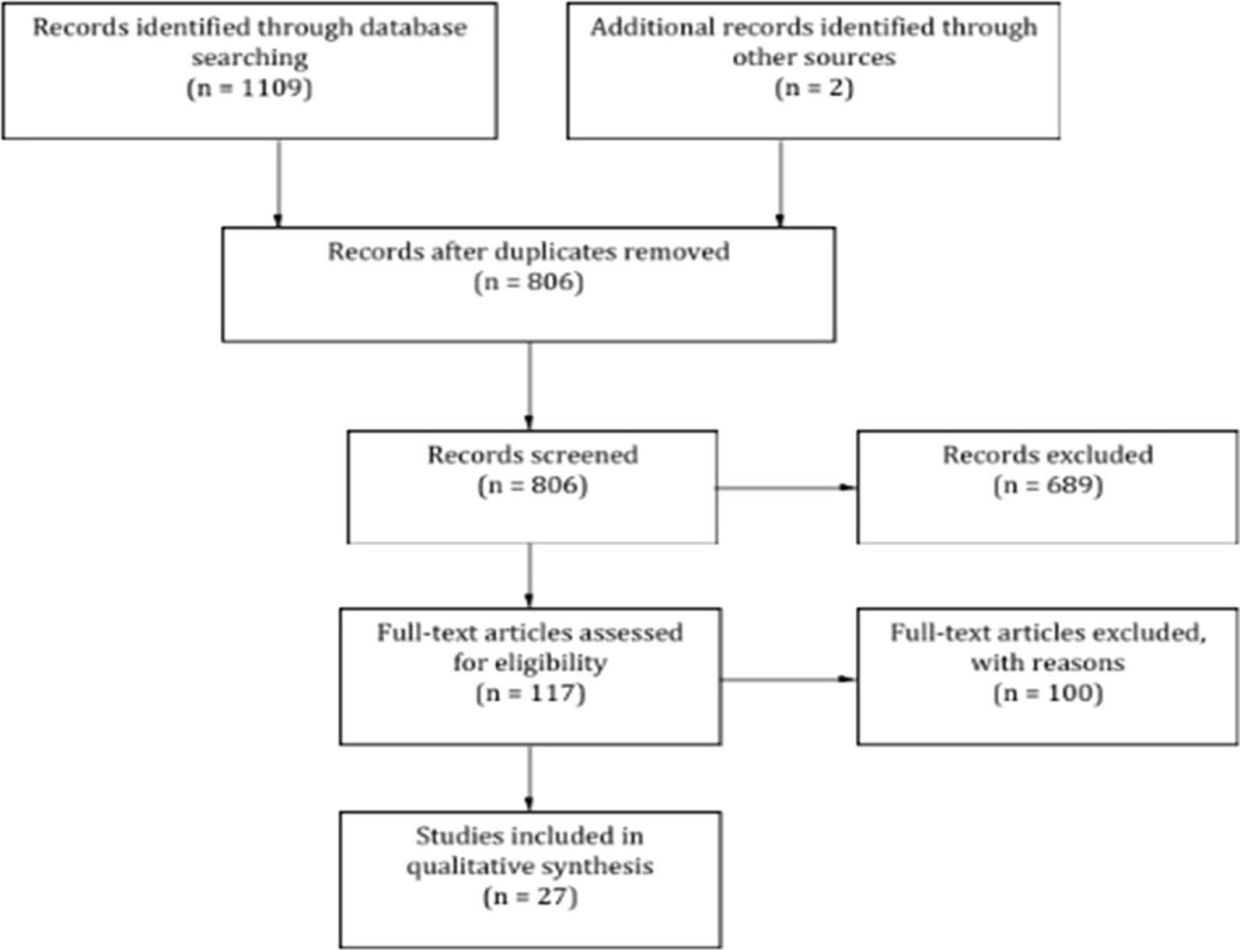
PRISMA flow diagram

Overall, 806 articles were identified through database searching. After screening the titles and abstract, 689 articles were excluded according to the exclusion criteria leaving 117 articles for full-text review. A further 100 articles were excluded after full text review and 27 articles remained to be included. A summary of the study selection process is shown in the PRISMA flow diagram (Fig. 1) [12, 22–44].

Temporal changes of haematological findings of the COVID-19 infection was estimated using their mean and SD for the corresponding data of onset. If mean and SD were not available, it was estimated those using median and quartiles [45]. When there are two are more mean readings from different studies for the same date of onset (DOO), a weighted average was computed and used for generating trend. Trend for the blood parameters by DOO were generated, only if the parameters were available for at least 3 DOO time points.

## Results

### Demographics

There were a total of 27 studies included in the final review. All of the studies were published in 2020 and were done in China. Of the 27 included studies, 25 were retrospective studies and 2 prospective studies [25, 33]. The number of patients included in the studies ranged from 10 to 248 with the mean age ranging from 40.0 to 72.5 years old. Overall, there were 2152 confirmed cases of COVID-19 patients included in our review with 49.1% were females. Most of the studies have an equal distribution of patients for both genders except for one that reported specifically on children and pregnant women [28].

For the studies that reported on the co-morbidities of their patients, the most common conditions included hypertension, hyperlipidemia, diabetes mellitus, lung diseases (such as chronic obstructive pulmonary disease), renal diseases, liver diseases, cerebrovascular diseases and tumours [22, 24–27, 30–32, 34–44, 46, 47]. Smoking status was also reported in eight studies [14, 16, 24, 25, 32, 36, 40, 44]. Furthermore, one study focused on critically ill patients [39] while another consisted of only hospitalized death patients [47]. The demographics of the included studies are presented in Table 1.

**Table 1:** Demographics

S/N		Total participants	Age in years (Mean ± SD) or Median (IQR)	Females	Comorbidities								Smoking status
					Hypertension	Diabetes Mellitus	Lung disease	Heart disease	Renal disease	Liver disease	Tumour	Cerebrovascular disease	
1	Zhang 2020	82	72.5 years (IQR 65.0–80.0)	28 (34.1%)	46 (56.1%)	15 (18.3%)	12 (14.6%)	17 (20.7%)	4 (4.9%)	2 (2.4%)	6 (7.3%)	10 (12.2%)	NR
2	Han 2020	108	Mean: 45 years (Range 21–90)	70 (64.8%)	NR	NR	NR	NR	NR	NR	NR	NR	NR
3	Zhang 2020	140	Median: 57 years (Range 25–87)	69 (49.3%)	42 (30.0%)	17 (12.1%)	4 (2.85%) COPD, pulmonary TB	14 (10.0%) CHD, arrhythmia, aorta sclerosis	2 (1.4%)	8 (5.7%)	NR	3 (2.1%)	9 (6.4%) total smokers 7 (5.0%) past smokers 2 (1.4%) current smokers
4	Shi 2020	81	(49.5 ± 11.0), range 25–81)	39 (48.1%)	12 (14.8%)	10 (12.3%)	9 (11.1%)	8 (9.88%)	3 (3.70%)	7 (8.64%)	4 (4.94%)	6 (7.41%)	NR
5	Huang 2020	41	Median: 49.0 (Range 41–58)	11 (26.8%)	6 (14.6%)	8 (19.5%)	1 (2.44%)	6 (14.6%)	NR	1 (2.44%)	1 (2.44%)	NR	3 (7.32%) current smokers
6	Pan 2020	21	(40 ± 9), range 25–63)	15 (71.4%)	NR	NR	NR	NR	NR	NR	NR	NR	NR
7	Chen 2020	249	51 (IQR 36–64)	123 (49.4%)	NR	NR	5 (2.0%)	55 (27.1%) Includes cerebrovascular disease	NR	2 (0.8%)	1 (0.4%)	NR	NR
8	Bernheim 2020	121	(45 ± 15.6), range 18–80)	60 (49.5%)	NR	NR	NR	NR	NR	NR	NR	NR	NR
9	Xiong 2020	42	(49.5 ± 14.1), range 26–75)	17 (40.5%)	NR	NR	NR	13 (31.0%)	NR	NR	NR	NR	NR
10	Wang 2020	138	56 (IQR 42–68), range 22–92)	63 (45.7%)	43 (31.2%)	14 (10.1%)	4 (2.9%)	20 (14.5%)	4 (2.9%)	4 (2.9%)	10 (7.2%)	7 (5.1%)	NR
11	Liu 2020	55	33.5 (27–58)/30 (26–35)/31 (22–42)	50 (90.9%)	NR	NR	NR	NR	NR	NR	NR	NR	NR
12	Wang 2020	90	45 ± 14	57 (63.3%)	NR	NR	NR	NR	NR	NR	NR	NR	NR
13	Yuan 2020	27	60 (IQR 47–69)	15 (55.6%)	5 (18.5%)	6 (22.2%)	NR	3 (11.1%)	NR	NR	1 (3.70%)	1 (3.70%)	NR
14	Xu 2020	62	41 (IQR 32–52)	27 (43.5%)	5 (8.06%)	1 (1.61%)	1 (1.61%)	NR	1 (1.61%)	7 (11.3%)	NR	1 (1.61%)	NR
15	Zhu 2020	32 COVID-19)	46 (IQR 35–52)	17 (53.1%)	7 (21.8%)	4 (12.5%)	2 (6.25%)	2 (6.25%)	1 (3.13%)	2 (6.25%)	2 (6.25%)	1 (3.13%)	6 (18.8%) current smokers
16	Zhou 2020	62	Early stage group, mean: 44.32 years (SD 13.53, range 20–72) Progressive stage group, mean: 50.82 years (SD 13.23, range 22–91)	Total: 28 (45.2%), early stage group: 16, progressive stage group: 12	NR	NR	NR	NR	NR	NR	NR	NR	NR
17	Song 2020	51	(49 ± 6–16), range 16–76)	26 (50.1%)	5 (9.80%)	3 (5.88%)	1 (1.96%)	1 (1.96%)	NR	1 (1.96%)	NR	NR	3 (5.88%) current smokers
18	Wang 2020	69	42.0 (IQR 35.0–62.0)	37 (53.6%)	9 (13.0%)	7 (10.1%)	6 (8.70%) COPD, asthma	8 (11.6%)	NR	1 (1.45%)	4 (5.80%)	NR	NR
19	Li 2020	78	44.6 ± 17.9	40 (51.3%)	10 (12.8%)	4 (5.1%)	9 (11.5%)	2 (2.6%)	NR	1 (1.3%)	3 (3.8%)	1 (1.3%)	5 (6.8%) current smokers 2 (2.6%) past smokers
20	Wu 2020	80	44 ± 11	38 (47.5%)	4 (5.0%)	4 (5.0%)	3 (3.75%)	1 (1.25%)	NR	NR	NR	NR	26 (33%) current smokers
21	Liu 2020	12	53.7 ± 17.2	4 (33.3%)	3 (25.0%)	2 (16.7%)	1 (8.33%)	4 (33.3%)	1 (8.33%)	NR	NR	NR	NR
22	Bai 2020	219 COVID-19)	(44.8 ± 14.5), range 4–76)	COVID-19 patients: 100 (45.7%)	31 (14.2%)	NR	9 (4.11%)	12 (5.48%)	2 (0.913%)	6 (2.74%)	3 (1.37%)	NR	NR
23	Liu 2020	10	42 (IQR 34–50)	6 (60.0%)	1 (10.0%)	NR	NR	NR	NR	1 (10.0%)	NR	NR	1 (10.0%) current smokers
24	Yang 2020	149	(45.1 ± 13.4)	68 (45.6%)	NR	NR	1 (0.67%)	28 (18.79%)	NR	NR	2 (1.34%)	NR	NR
25	Yang 2020	52	Survivors, mean: 51.9 years (SD 12.9) Non-survivors, mean: 64.6 years (SD 11.2) All patients: 59.7 years (SD 13.3)	Survivors: 6 (30%) Non-survivors: 11 (34.4%) All patients: 17 (32.7%)	NR	9 (17.3%)	4 (7.69%)	5 (9.62%)	NR	NR	2 (3.85%)	7 (13.5%)	2 (3.85%) current smokers
26	Zhao 2020	19	48 (IQR 27–56)	8 (42.1%)	2 (10.5%)	NR	NR	NR	NR	NR	NR	NR	NR
27	Zhou 2020	62	(52.8 ± 12.2), range, 30–77)	23 (37.1%)	4 (6.45%)	4 (6.45%)	NR	NR	1 (1.61%)	NR	NR	1 (1.61%)	NR

*NR* not reported

Out of the 27 studies, 6 of them reported purely clinical laboratory findings, 11 reported purely CT imaging findings while 10 reported both findings.

### Haematological findings

Common laboratory findings that were reported included lymphopenia, elevated lactate dehydrogenase (LDH) levels and elevated inflammatory markers such as C-reactive protein (CRP). Hence our analysis primarily focussed on these four parameters. From our review, a total of 15 studies reported lymphocyte counts, 11 reported neutrophil counts, 12 reported CRP levels and 11 reported LDH levels. The peripheral blood investigation results are summarised in Additional file 3.

Across the 15 studies that reported lymphocyte counts, the median ranged from 0.5 to 1.21 × 10^9^/L while the mean ranged from 0.62 to 1.4 × 10^9^/L. Eight studies reported medians or means that are suggestive of lymphopenia.

For neutrophil counts, the median ranged from 2.35 to 12.9 × 10^9^/L and the mean ranged from 0.71 to 3.1 × 10^9^/L. Only one study reported a neutrophil count with a mean that suggested neutropenia. The median CRP levels ranges from 11.7 to 84.9 mg/L with its mean ranging from 7.25 to 71.3, while the median LDH levels ranged from 194.5 to 784 U/L with its mean ranging from 210 to 246.5 U/L.

### Temporal progression of haematological findings

Out of the 16 publications that reported clinical laboratory findings, 12 reported blood investigations that were done on or before the seventh day since symptom onset (date of onset, DOO ≤ 7 days). Lymphocyte counts were reported in all 12 of the studies, 9 reported neutrophil counts, 9 reported CRP levels and 9 reported LDH levels. Blood investigations done after the seventh day since symptom onset (DOO > 7 days) were reported in 5 studies; 3 reported lymphocyte counts, 2 reported neutrophil counts, 3 reported CRP levels and 2 reported LDH levels.

Furthermore, we compared the haematological and radiological findings between patients with severe and non-severe outcomes (Additional file 4) and analysed the temporal changes of blood investigations in patients stratified by the severity of patients’ outcome (Additional file 5).

**Fig. 2:**
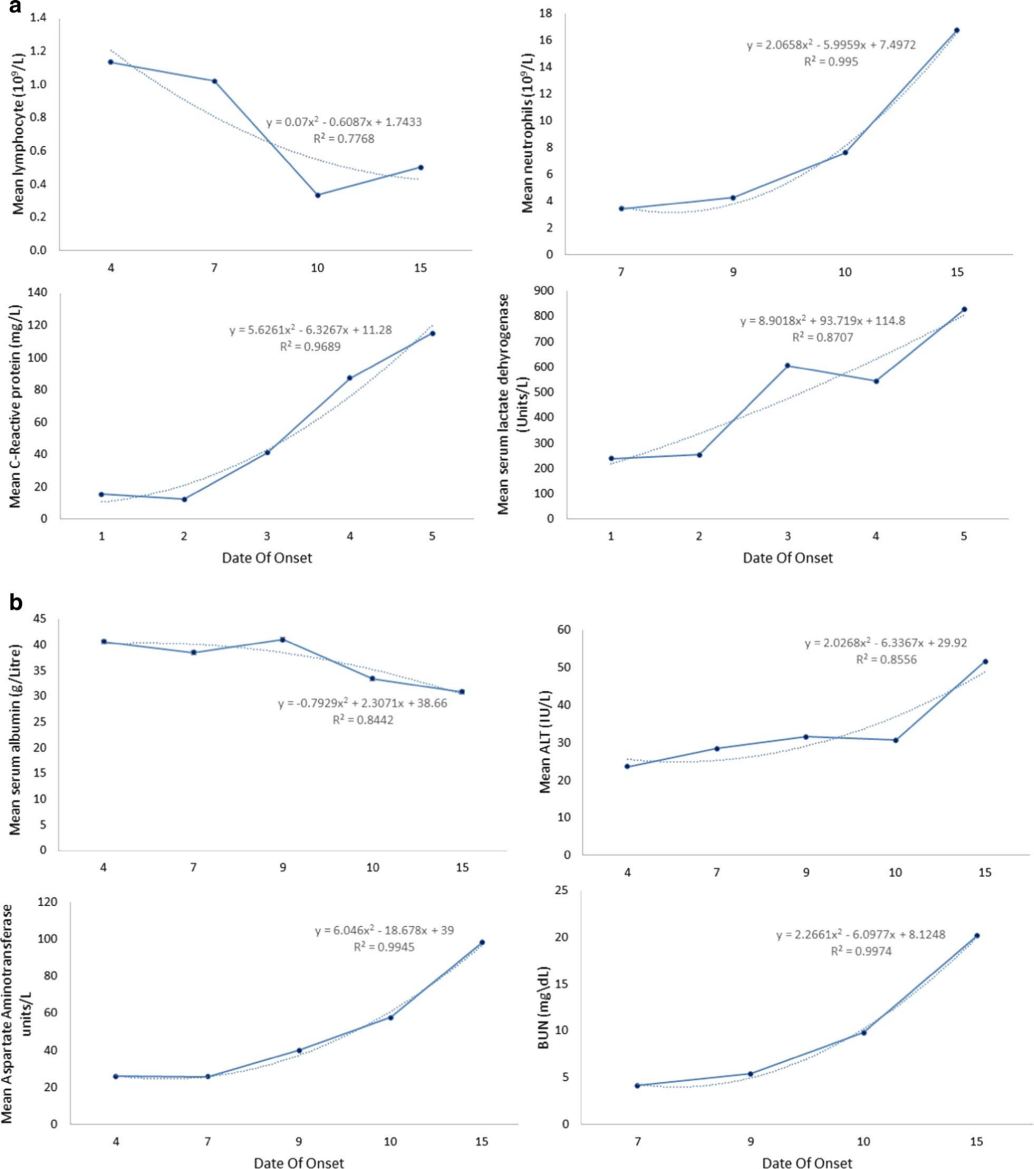
Temporal trends in haematological findings

Lymphopenia was evident in most studies regardless of the number of days since onset of symptoms. Mean lymphocyte count was lower for patients with DOO > 7 days compared to patients with DOO ≤ 7 days, 0.42 ± 0.30 versus 1.0 ± 0.49, *p* < 0.001, Table 2. The median for the lymphocyte count in patients with DOO ≤ 7 days ranged from 0.8 to 1.21 × 10^9^/L (mean range 0.97–1.4 × 10^9^/L). From the means and medians, lymphopenia were seen in 7 of the studies. For patients with DOO > 7 days, the median ranged from 0.5 to 0.9 × 10^9^/L (mean range 0.62–1.1 × 10^9^/L), with all but 1 study reporting medians that were suggestive of lymphopenia. Furthermore, all the studies regardless of the number of days since onset of symptoms, the reported medians or means of the lymphocyte counts were in the lower limit of normal. When stratified into patients with or without severe outcomes, 5 studies reported that the group with severe outcomes was associated with significantly lower levels of lymphocyte counts compared to the group without severe outcomes. Three papers also reported the temporal progression of lymphocyte counts in patients with severe outcomes and showed that the group with severe outcomes developed more severe lymphopenia over time. On the other hand, the lymphocyte percentage for the group without severe outcomes was monitored in one paper and was reported to have increased levels in a subsequent test. Temporal trend by DOO showed a polynomial decrease in mean lymphocyte level, Fig. 2a,b.

**Table 2:** Haematological findings by DOO

Variables	n	Weighted Mean ± SD	n	Weighted Mean ± SD	*P* value^a^
		DOO ≤ 7		DOO > 7	
Albumin	439	38.11 ± 4.91	176	35.09 ± 4.07	< 0.0001
Alanine aminotransferase	577	26.17 ± 18.55	176	37.98 ± 31.48	< 0.0001
Activated partial thromboplastin time	328	31.05 ± 5.22	164	36.07 ± 17.58	< 0.0001
Aspartate aminotransferase	577	30.08 ± 15.64	176	65.22 ± 54.83	< 0.0001
Bilirubin	328	10.79 ± 4.53	94	12.22 ± 5.79	0.0002
NT-proB-type natriuretic peptide			164	159.60 ± 12.71	
Blood urea nitrogen	287	4.17 ± 1.60	176	11.78 ± 9.15	< 0.0001
C3	NR	NR	82	0.93 ± 0.23	
C4	NR	NR	82	0.23 ± 0.08	
CD16/CD56	NR	NR	82	18.77 ± 12.00	
CD19	NR	NR	82	17.83 ± 11.47	
CD3	NR	NR	82	119.47 ± 16.75	
CD4	249	455.7 ± 252.0	82	33.67 ± 12.15	< 0.0001
CD8	NR	NR	82	17.97 ± 11.77	
Creatine kinase	328	102.33 ± 76.52	164	238.00 ± 256.76	< 0.0001
Creatinine kinase MB	138	14.00 ± 5.99	164	3.82 ± 3.17	< 0.0001
Creatinine	328	70.51 ± 18.89	176	119.31 ± 98.56	< 0.0001
C-Reactive protein	478	14.81 ± 25.93	176	81.10 ± 68.26	< 0.0001
Ddimer	328	81.08 ± 70.77	164	25.90 ± 31.47	< 0.0001
eGFR	249	110.6 ± 56.1	NR	NR	
ESR	249	59 ± 42.5	NR	NR	
Haemoglobin	41	128.0 ± 16.9	NR	NR	
IGA	NR	NR	82	2.73 ± 1.36	
IGE	NR	NR	82	81.07 ± 97.57	
IGG	NR	NR	82	13.50 ± 4.53	
IGM	NR	NR	82	0.97 ± 0.45	
Interleukin 6	NR	NR	164	173.72 ± 132.17	
Lactate	249	1.53 ± 0.75	NR	NR	
Lactic acid dehydrogenase	577	260.58 ± 114.80	176	659.33 ± 363.56	< 0.0001
Lymphocyte	657	1.02 ± 0.49	164	0.42 ± 0.30	< 0.0001
Myoglobin	NR	NR	176	252.30 ± 331.41	
Neutrophil	408	3.88 ± 2.60	176	9.53 ± 6.21	< 0.0001
PCo2	NR	NR	82	34.00 ± 10.56	
pH	NR	NR	82	7.23 ± 0.30	
Platelet	328	173.28 ± 76.74	176	137.17 ± 63.94	< 0.0001
Potassium	41	4.27 ± 0.77	164	4.18 ± 0.42	0.3119
Procalcitonin	121	0.07 ± 0.02	164	1.57 ± 2.57	< 0.0001
Prothrombin time	328	12.13 ± 1.45	164	16.35 ± 5.47	< 0.0001
Sodium	41	138.67 ± 2.30	164	144.92 ± 7.36	< 0.0001
Troponin	138	9.23 ± 11.76	12	0.96 ± 3.14	0.0167
Troponin T	NR	NR	164	1.73 ± 3.47	
White blood cells	657	5.29 ± 2.64	12	5.97 ± 17.20	0.4958

*NR* not reported

^a^Independent samples *t* test

In terms of neutrophil count, it was mostly normal for both DOO ≤ 7 days and DOO > 7 days. However, mean neutrophil count was significantly higher for papers that reported DOO > 7 days compared to those that reported DOO ≤ 7 days, 9.53 ± 6.21 versus 3.88 ± 2.60, *p* < 0.0001, Table 2. The range of median of neutrophil count for DOO ≤ 7 days was 2.35–5.0 × 10^9^/L (mean range 0.71–3.1 × 10^9^/L) while the median range for DOO > 7 days was 3.36–12.9 × 10^9^/L. Across the 11 studies that reported on neutrophil counts, only one study reported a mean that is suggestive of neutrophilia for DOO ≤ 7 days. However, there were three studies in which there were higher neutrophil counts in the group with severe outcomes as compared to the group without severe outcomes. Temporal trend by DOO showed a polynomial increase in mean neutrophil level, Fig. 2a,b.

Out of all the studies that reported CRP levels, all but one reported elevated CRP levels regardless of the number of days since onset of symptoms. Mean CRP levels were significantly higher for papers that reported DOO > 7 days compared those that reported DOO ≤ 7 days, 14.81 ± 25.93 versus 81.10 ± 68.26, *p* < 0.001, Table 2. The median CRP levels for publications that reported DOO ≤ 7 days ranged from 12 to 35.62 mg/L (mean range 7.25–61.4 mg/L). The median and mean CRP levels that were reported for DOO > 7 days were all elevated; the median range for DOO > 7 days were 11.7–84.9 mg/L (mean range 41.1–71.3 mg/L). In the five studies that reported CRP levels and divided their patients into those with severe outcomes and those without, CRP levels were more prominently elevated in the former group for all of them. Temporal trend by DOO showed a polynomial increase in mean CRP level, Fig. 2a,b.

Similarly, LDH levels were mostly elevated. However, the LDH levels for the papers that reported DOO > 7 days were significantly more elevated than those reported DOO ≤ 7 days, 659.33 ± 363.56 versus 260.58 ± 114.80, *p* < 0.0001, Table 2. For DOO ≤ 7 days, the median for LDH levels ranged from 194.5 to 286 U/L (mean range 210–246.5 U/L) while the LDH median range for DOO > 7 days was 515–784 U/L. In fact, LDH levels were positively correlated to the severity of patients’ outcomes as reported in five of the studies. Temporal trend by DOO showed a polynomial increase in mean LDH, Fig. 2a,b. Besides this, other haematological parameters such as aspartate aminotransferase, bilirubin, blood urea nitrogen (BUN), creatinine kinase, creatinine, procalcitonin, prothrombin time and sodium were significantly higher for DOO > 7 compared to DOO ≤ 7. While it was significantly lower for albumin, alanine amino transferase, CD4, creatinine kinase (MB), d-dimer, platelets, potassium, and troponin for DOO > 7 compared to DOO ≤ 7, Table 2a,b.

### CT imaging findings

Only studies which state the mean duration of the scans from date of symptom onset and those which report specific lung findings are included in our analysis. A total of 21 studies are included in our analysis, with 12 studies reporting findings ≤ 7 days from symptom onset, 3 studies reporting findings > 7 days from symptom onset, and 6 studies reporting findings from both groups. Certain chest CT image features are commonly reported across the various studies and these features are summarised in Table 3.

**Table 3:** Common chest CT imaging features across 21 studies

CT signs	Imaging finding	Number of studies	No. of reported cases (%)/Total no. of cases	Yang 2020 (S/N 24) (by lung segments)
Lung changes	GGO	16	1056/1610 (65.6%)	287/2376 (12.1%)
	Consolidation	17	462/1325 (34.9%)	170/2376 (7.15%)
	GGO + Consolidation	5	135/293 (46.1%)	637/2376 (26.8%)
	Crazy paving pattern	11	280/1064 (26.3%)	NR
Lesion distribution	Bilateral lungs	13	1239/1568 (79.0%)	NR
	Unilateral lung	9	161/1187 (13.6%)	NR
	No of lobes: 1	6	82/455 (18.0%)	NR
	No of lobes: 2 or 3	6	92/455 (20.2%)	NR
	No of lobes: 4 or 5	6	226/455 (49.7%)	NR
	Peripheral	12	862/1250 (69.0%)	853/2376 (35.9%)
	Central	12	51/1148 (4.44%)	51/2376 (2.15%)
	Peripheral + Central	8	175/652 (26.8%)	193/2376 (8.12%)

*NR* not reported

The commonly reported CT features of COVID-19 pneumonia include lung changes such as ground glass opacities (GGO), consolidation, GGO plus consolidation, and crazy paving pattern, as well as lesion distribution such as bilateral or unilateral lung involvement, number of lobes involved, and whether the lesions are distributed peripherally, centrally or both peripherally and centrally. The main CT patterns were described in line with the terms defined by the Fleischner Society and peer-reviewed literature on viral pneumonia.

In terms of lung changes, 16 studies showed that 1056/1610 scans (65.6%) had GGO, 17 studies showed that 462/1325 scans (34.9%) had consolidation, 5 studies showed that 135/293 scans (46.1%) had both GGO and consolidation, and 11 studies showed that 280/1064 scans (26.3%) had crazy paving pattern.

In terms of lesion distribution, 13 studies showed that 1239/1568 scans (79.0%) had bilateral lung involvement while 9 scans showed that 161/1187 scans (13.6%) had unilateral lung involvement. 6 studies reported that only 1 lobe was involved for 82/455 scans (18.0%), 2 or 3 lobes were involved for 92/455 scans (20.2%), and 4 or 5 lobes were involved for 226/455 scans (49.7%). 12 studies showed that 862/1250 scans (69.0%) had the lesions distributed peripherally, while 51/1148 scans (4.44%) had lesions distributed centrally. 8 studies showed that 175/652 scans (26.8%) had lesions distributed both peripherally and centrally.

We separately analysed a study by Yang et al. [38] as the lung changes were reported by the number of lung segments instead of the number of scans or patients. In this case, the study showed that 287/2376 segments (12.1%) presented GGO, 170/2376 segments (7.15%) presented consolidation, and 637/2376 segments (26.8%) presented both GGO and consolidation. In terms of the lesion distribution, the study showed that the lesions were more localized in the periphery rather than the center of the lung (853, 35.9% vs 51, 2.15%).

While considering how chest CT findings may predict outcome severity, we analysed 5 studies which discussed findings from groups with severe outcomes compared to groups with non-severe outcomes (Additional file 4). From all 5 studies, the group with severe outcomes typically exhibit a greater degree and frequency of consolidation, more extensive lesion distribution, and a higher frequency of multilobe bilateral lung involvement. One study [30] even reported incidences of “white lungs” on CT imaging of the most severely affected patients.

### Temporal progression of CT imaging findings

When the course of disease was divided into an early phase (≤ 7 days after the onset of symptoms) and a later phase (> 7 days after the onset of symptoms), we found several interesting trends. The findings are summarised in Additional files 6 and 7 for the early phase and later phase, respectively. The comparison of the various CT imaging findings between the early and later phases is summarised in Table 4.

**Table 4:** Comparison between early and late phase chest CT findings

	Lung changes				Lesion distribution							
	GGO	Consolidation	GGO + Consolidation	Crazy paving pattern	Bilateral lungs	Unilateral lung	No of lobes: 1	No of lobes: 2 or 3	No of lobes: 4 or 5	Peripheral	Central	Peripheral + Central
Early phase	827/1108 (74.6%)	394/1053 (37.4%)	157/317 (49.5%)	191/872 (21.9%)	800/1042 (76.8%)	131/814 (16.1%)	95/444 (21.4%)	83/403 (20.6%)	188/403 (46.7%)	506/698 (72.5%)	49/720 (6.81%)	109/518 (21.0%)
Late phase	164/292 (56.2%)	126/287 (43.9%)	8/27 (29.6%)	76/265 (28.7%)	423/461 (91.8%)	23/207 (11.1%)	11/108 (10.2%)	6/67 (8.96%)	56/67 (83.6%)	52/139 (37.4%)	3/139 (2.16%)	77/114 (67.5%)

*NR* not reported

In terms of the lung changes, the frequency of GGO was higher in early-phase disease (827/1108, 74.6%) than in later-phase disease (164/292, 56.2%). A similar relationship is seen for the frequency of GGO with consolidation; early phase (157/317, 49.5%) versus later phase (8/27, 29.6%). However, the frequency of consolidation was lower in early-phase disease (394/1053, 37.4%) than in later-phase disease (126/287, 43.9%). This trend is also reflected in the frequency of crazy paving pattern; early phase (191/872, 21.9%) versus later phase (76/265, 28.7%).

In terms of lesion distribution, there is a lower frequency of bilateral lung involvement in early-phase disease (800/1042, 76.8%) as compared to that in later-phase disease (423/461, 91.8%). Logically, the converse is seen for the frequency of unilateral lung involvement; early-phase (131/814, 16.1%) vs later-phase (23/207, 11.1%). For the number of lobes involved, the frequency for 4–5 lobar involvement is highest within either of the early and later phase diseases respectively: early-phase (188/403, 46.7%), later-phase (56/67, 83.6%). Comparing the early-phase to the later-phase, there is a higher frequency of 4–5 lobar involvement in the later-phase (56/67, 83.6%) compared to the early-phase (188/403, 46.7%). In early-phase disease, there appears to be a higher predilection for the peripheries (506/698, 72.5%), whereas in later-phase disease, the lesions appear to be distributed both peripherally and centrally with highest frequency (77/114, 67.5%).

One study [40] compared the temporal changes of CT findings between their subgroup of patients who survived and those who did not (Additional file 5). They found that for the survival group, when re-examination chest CT findings were compared those on admission, the morphology of the lesions, locations, extents, and distribution of involvement of each abnormality were not significantly changed. This contrasts with the mortality group, for which the CT scores progressed rapidly in a short time.

### Haematological and radiological features of asymptomatic patients

We also attempted to look at the laboratory and radiological features of asymptomatic patients with confirmed diagnosis of COVID-19. One study [30] specifically reported on asymptomatic patients (n = 15) with confirmed exposure history who were eventually diagnosed with COVID-19 pneumonia. Shi et al. [30] found that these patients had significantly lower mean concentrations of CRP and aspartate aminotransferase (AST) than patients after onset of symptoms. There was no significant difference in the other laboratory findings such as leukocyte and lymphocyte counts. In terms of radiological findings, these asymptomatic patients all had abnormal CT imaging features albeit with less extensive lung involvement compared to symptomatic patients. The typical pattern seen in CT from the subclinical patients comprised unilateral (nine [60%] patients) and multifocal (eight [53%]) ground-glass opacifications (14 [93%]). These lesions rapidly evolved to bilateral and diffuse ground-glass opacifications after symptom onset.

## Discussion

In patients diagnosed with COVID-19, laboratory findings that were commonly present included lymphopenia, raised CRP levels and raised LDH levels. By comparing the peripheral blood investigation findings between the earlier phase (DOO ≤ 7 days) and the later phase (DOO > 7 days) of the disease, there was more prominent lymphopenia and raised inflammatory markers like CRP and LDH levels in the latter. Neutrophil counts were higher in the later phase compared to the earlier phase as well.

Regardless of the number of days since onset of symptoms, lymphopenia can be seen in most hospitalised patients with COVID-19. Besides studies from China, studies from Saudi Arabia [48], Iran [49], India [50], South Korea [51], Singapore [52] and cruise ship (Diamond princess) [53] have also confirmed this finding However, the extent of lymphopenia is more prominent in the early phase (DOO ≤ 7 days) compared to those in the later phase (DOO > 7 days). It has been hypothesized that lymphopenia could be due to direct infection of the lymphocyte by the virus, destruction of lymphatic organs, lymphocyte apoptosis due to inflammation or inhibition of lymphocytes due to metabolic disorders like lactic acidosis [54, 55]. As such, we postulate that at a later timing as the disease progresses, more severe metabolic processes could further inhibit lymphocytes. Furthermore, worsening lymphopenia is also evident in the clinical course of patients with severe outcomes compared to those without. As such, the progression of lymphopenia in COVID-19 patients would be a good predictor of severity and outcomes. Since disease progression is associated with worsening lymphopenia and hence severity, lymphocyte counts could be employed as a tool for prognostication in terms of the severity of the disease especially for patients in the later phase of the disease [11].

Elevations in biomarkers such as CRP and LDH levels were also more significant in the later phase of the disease (DOO > 7 days) than the earlier phase (DOO ≤ 7 days). Similar elevation in CRP was also observed among patients in Saudi Arabia [48] and Iran [49] and was associated with mortality. CRP, an acute phase reactant, increases rapidly after the onset of inflammation, cell damage or organ injury in response to inflammatory cytokines [56]. Similarly, LDH is released from cells upon damage of their cytoplasmic membrane [57]. As such, a pulmonary disease such as COVID-19 would cause an increase in these biomarkers. At a later phase, even higher levels of CRP and LDH could be a manifestation of disease progression as a result of the organ damage due to direct attacks from the virus causing excessive inflammatory response. Given that these biomarkers correlate positively to the disease progression, it may also be useful to monitor severity in patients.

Neutrophil counts in the later phase (DOO > 7 days) were also noted to be higher than those in the earlier phase (DOO ≤ 7 days). Similar phenomenon was observed in Singapore [52] and Iran [49]. The increase in neutrophil count as the disease progresses was thought to be due to the sustained virus invasion leading to a cytokine storm [32]. Furthermore, patients with severe outcomes have a more prominent elevation in neutrophil counts than those without severe outcome. Thus, an increase in neutrophil count could be associated with disease progression and severity. This would be beneficial as neutrophil counts could then be used to monitor the severity and disease progression in patients with COVID-19.

Overall, the most commonly reported lung feature from our analysis is GGO (1056/1610 scans, 65.6%), followed by GGO with consolidation (135/293 scans, 46.1%), then consolidation (462/1325 scans, 34.9%), and finally crazy paving pattern (280/1064 scans, 26.3%). Similar patterns were observed in studies elsewhere [50, 53, 58–60]. These CT findings are likely related to the complex pathological changes in the lungs of patients with COVID-19. Tian et al. [61] reported that histologically, the main findings in the lungs include injury to the alveolar epithelial cells, hyaline membrane formation, and hyperplasia of type II pneumocytes, all of which are components of diffuse alveolar damage. Consolidation by fibroblastic proliferation with extracellular matrix and fibrin forming clusters in airspaces is evident. Zhe et al. [62] also concurred, reporting that there is diffuse alveolar damage with cellular fibromyxoid exudates, as well as desquamation of pneumocytes and hyaline membrane formation, indicating acute respiratory distress syndrome (ARDS). These pathological changes may be the main pathological basis of the CT findings.

From our analysis, the frequency of GGO was higher in early-phase disease than in later-phase disease. Pathologic examinations reported by Tian et al. [61] found that edema, proteinaceous exudate, focal reactive hyperplasia of pneumocytes with patchy inflammatory cellular infiltration, and multinucleated giant cells were all present in the early phase of the disease. However, in later-phase disease, the frequency of consolidation and that for crazy paving pattern were higher than those in early-phase disease. We postulate that this might be due to further infiltration of the lung parenchyma and they are also typical features of the underlying pathophysiology of an organising pneumonia [63]. Also, consolidation is considered as an indication of disease progression and crazy paving pattern could be the signal of COVID-19 entering progressive or peak stage over time [64].

In terms of lesion distribution, the SARS-CoV-2 virus seems to cause a significantly higher frequency of bilateral lung involvement compared to unilateral, and this is even more so in the later phase. Multilobe involvement also seems to be a prominent feature, with 4–5 lobe involvement occurring at the highest frequency. This pattern is further evident in the later phase. Overall, the lesions seem to be mostly distributed peripherally and this is also the case in early-phase disease. However, in later-phase disease, the lesions appear to be distributed both peripherally and centrally with highest frequency. This predominantly bilateral, multilobar and peripheral lung distribution concurs with other radiological studies [65] on the virus and appears to be a hallmark chest CT feature [65]. This may suggest a high virulence factor of the SARS-CoV-2 virus due to its extensive penetration of the lung parenchyma [66].

All these temporal changes on chest CT imaging suggest the utility of this investigation modality in monitoring for disease progression in COVID-19 patients, and this is in agreement with the consensus statement from the Fleischner Society [67].

Our analysis has also demonstrated key differences in chest CT findings between groups with severe outcomes and those with non-severe outcomes. The former typically exhibits a greater degree and frequency of consolidation, more extensive lesion distribution, and a higher frequency of multilobe bilateral lung involvement. Temporally, the lung involvement on CT of the former also progressed more extensively and rapidly, while no significant changes were noted over time for the groups with non-severe outcomes. Additionally, the worsening radiological features coincide with the worsening of lymphopenia, neutrophilia, CRP elevation and LDH elevation which signify the worsening of systemic inflammation and viral invasion. These all suggest that such CT findings could help predict and identify patients at risk of severe clinical outcomes.

This review is included 27 studies, all of which were conducted in China. Also, amongst the included studies, only one study was conducted among critical ill patients. This is a limitation as the number of COVID-19 cases and deaths in many other countries including the United States and several European countries have since exceeded that of China [7]. However, our review findings are similar to emerging evidence of haematological and radiological from other parts of the world [48–53, 58–60, 68].

Majority of the studies (n = 25) were retrospective and thus had inherent design-specific limitations such as bias in selection, residual confounding, and temporal ambiguity. Some studies were unable to be included in the evaluation of infection time course due to the lack of precise date of symptom onset reported. Furthermore, the number of studies that reported findings on COVID-19 patients in the later phase (DOO > 7 days) was also deficient. In addition, there were few studies included in this review that monitored the clinical course of a patient from symptom onset to their ultimate outcome, which would allow for better analysis of clinical progression of either the peripheral blood investigations or radiological investigations. Also, different normal ranges for the peripheral blood investigation results were reported in different studies which made comparison difficult, particularly when trying to account for slight elevations or decreases in the results, which at an early phase may herald disease progression. Furthermore, this review did not account for the treatments administered to the patients in the various studies, which include antivirals, corticosteroids, antimicrobials and oxygen therapy among others. Although there is currently still no reliable and effective treatment for COVID-19 to date, the various medical interventions may have altered the natural progression of the disease. Hence, the temporal changes of both the peripheral blood and CT imaging findings may not be the most reliable and applicable to other patient populations.

As such, further reviews can be conducted to address the above limitations of this review, particularly because many studies on COVID-19 patients in countries besides China have since been published.

More studies in the outpatient, primary care, or community settings are also needed to get a full picture of the spectrum of clinical severity. Additional effort can be also made to conduct a similar study in other patient populations, such as the paediatric and adolescent age groups. Further studies can be done to distinguish between the laboratory and radiological manifestations of COVID-19 and the rest of its viral family, particularly SARS and MERS, as well as with other kinds of viral pneumonia such as influenza virus, parainfluenza virus, adenovirus, respiratory syncytial virus, rhinovirus, human metapneumovirus, and mycoplasma pneumonia.

## Conclusion

With time progression, peripheral blood results reflect worsening lymphopenia, more significant elevations in CRP and LDH as well as that for neutrophil counts. Temporal change also shows decrement of the frequency of GGO, as opposed to increasing consolidation and crazy paving patterns on chest CT imaging. The lesion distribution in early-phase disease already appears extensive and continues causing more lung involvement over time. These findings suggest a positive correlation between temporal progression, disease severity and the reported blood and radiology results. Awareness of these trends in blood results and imaging changes may assist clinicians in making ICU admission decisions when resources are limited.

## Supplementary Information

**Additional file 1.** Search strategy.

**Additional file 2.** Sample of data extraction table.

**Additional file 3.** Peripheral blood investigation results. NR = Not reported.

**Additional file 4.** Comparison of blood and radiology findings between severe and non-severe patients. NR = not reported.

**Additional file 5.** Temporal changes of blood and radiology results stratiied by patients' outcocmes. NR = not reported.

**Additional file 6.** Chest CT imaging findings for early phase. NR = not reported.

**Additional file 7.** Chest CT imaging findings for late phase. NR = not reported.

## Data Availability

The datasets used and/or analysed during the current study are available from the corresponding author on reasonable request.

## Abbreviations

WHO: World Health Organisation
COVID-19: Coronavirus disease-19
SARS-CoV-2: Severe acute respiratory syndrome coronavirus 2
SARS: Severe acute respiratory syndrome
MERS: Middle East respiratory syndrome
ARDS: Acute respiratory distress syndrome
PRISMA: Preferred Reporting Items for Systematic Reviews and Meta-Analyses
MeSH: Medical Subject Headings
CT: Computed tomography
LDH: Lactate dehydrogenase
CRP: C-reactive protein
DOO: Date of onset
GGO: Ground glass opacities
