# Supplementary figures and images for: Temporal changes of haematological and radiological findings of the COVID-19 infection—a review of literature
Source: BMC Pulm Med. 2021 Jan 22;21:37. doi: 10.1186/s12890-020-01389-z (PMC7820529; doi:10.1186/s12890-020-01389-z)

*Additional file 1. Search strategy*


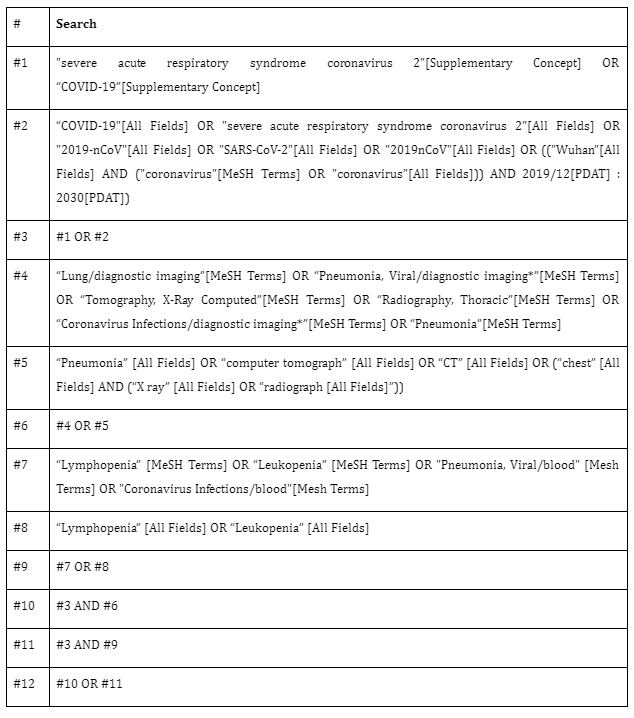

Supplement: Supplementary file 1 — Additional file 1. Search strategy. [file 12890_2020_1389_MOESM1_ESM.docx]
