# Supplementary material for: Temporal changes of haematological and radiological findings of the COVID-19 infection—a review of literature
Source: BMC Pulm Med. 2021 Jan 22;21:37. doi: 10.1186/s12890-020-01389-z (PMC7820529; doi:10.1186/s12890-020-01389-z)
Supplement: Supplementary file 2 — Additional file 2. Sample of data extraction table. [file 12890_2020_1389_MOESM2_ESM.docx]

*Additional file 2. Sample of data extraction table*

|  |  | **PAPER** | | | | | | | **COHORT** | | | | | **DEMOGRAPHICS** | | | | | **RADIOLOGY** | | | |
| --- | --- | --- | --- | --- | --- | --- | --- | --- | --- | --- | --- | --- | --- | --- | --- | --- | --- | --- | --- | --- | --- | --- |
| **S/N** | **CODE** | **PMID** | **AUTHORS** | **YEAR** | **TITLE** | **JOURNAL** | **TYPE** | **STUDY AIMS** | **COUNTRY** | **DATA SOURCE** | **DURATION OF STUDY** | **INCLUSION** | **EXCLUSION** | **TOTAL PARTICIPANTS(T)** | **AGE in mean (SD)  *include the range if given** | **FEMALE** | **MALE** | **OTHERS/COMORBIDS** | **CXR?** | **CXR FINDINGS** | **CT?** | **CT FINDINGS** |
|  |  |  |  |  |  |  |  |  |  |  |  |  |  |  |  |  |  |  |  |  |  |  |
|  |  |  |  |  |  |  |  |  |  |  |  |  |  |  |  |  |  |  |  |  |  |  |

| **BLOOD DATA** | | | | | | | | | | | | | | | | | | | | | | | | | | |
| --- | --- | --- | --- | --- | --- | --- | --- | --- | --- | --- | --- | --- | --- | --- | --- | --- | --- | --- | --- | --- | --- | --- | --- | --- | --- | --- |
| **FBC: HEMOGLOBIN (g/L)** | **FBC: TOTAL WHITE COUNT (× 10^9 /L)** | **FBC: NEUTROPHIL COUNT (× 10^9 /L)** | **FBC: LYMPHOCYTE COUNT(× 10^9 /L)** | **FBC: MONOCYTE COUNT (× 10^9 /L)** | **FBC: EOSINOPHIL COUNT (× 10^9 /L)** | **FBC: PLATELET COUNT (× 10^9 /L)** | **CRP (mg/L)** | **LFT: ALT (U/L)** | **LFT: AST (U/L)** | **LFT: TOTAL BILIRUBIN (µmol L–1)** | **LFT: ALBUMIN (g/L)** | **RP: POTASSIUM (mmol/L)** | **RP: SODIUM (mmol/L)** | **RP: BLOOD UREA NITROGEN (mmol/L)** | **RP: CREATININE (μmol/L)** | **CREATININE KINASE (U/L)** | **MYOGLOBIN (ng/mL)** | **LDH (U/L)** | **CREATININE KINASE-MB (ng mL–1)** | **NT-PRO BNP** | **CARDIAC TROPONIN T** | **PROCALCITONIN (μg/L)** | **PT (s)** | **aPTT (s)** | **D-DIMER (mg/L)** | **OTHERS** |
|  |  |  |  |  |  |  |  |  |  |  |  |  |  |  |  |  |  |  |  |  |  |  |  |  |  |  |
|  |  |  |  |  |  |  |  |  |  |  |  |  |  |  |  |  |  |  |  |  |  |  |  |  |  |  |
