# Supplementary material for: Temporal changes of haematological and radiological findings of the COVID-19 infection—a review of literature
Source: BMC Pulm Med. 2021 Jan 22;21:37. doi: 10.1186/s12890-020-01389-z (PMC7820529; doi:10.1186/s12890-020-01389-z)
Supplement: Supplementary file 3 — Additional file 3. Peripheral blood investigation results. NR = Not reported. [file 12890_2020_1389_MOESM3_ESM.docx]

*Additional file 3. Peripheral blood investigation results. NR = not reported.*

|  |  | **Specific DOO** | **FBC: NEUTROPHIL COUNT (× 10^9 /L)** | | **FBC: LYMPHOCYTE COUNT(× 10^9 /L)** | | **CRP (mg/L)** | | **LDH (U/L)** | |  |
| --- | --- | --- | --- | --- | --- | --- | --- | --- | --- | --- | --- |
|  |  |  | **DOO ≤7 days** | **DOO >7 days** | **DOO ≤7 days** | **DOO >7 days** | **DOO ≤7 days** | **DOO >7 days** | **DOO ≤7 days** | **DOO >7 days** |  |
| 1 | Hamer 2020 | NR | NR | NR | NR | NR | NR | NR | NR | NR | |
| 2 | Guan 2020 | NR | NR | NR | NR | NR | NR | NR | NR | NR | |
| 3 | Zhang 2020 | 10 | NR | 6.8 (IQR 4.5-11.5) | NR | 0.5 (IQR 0.3-0.8) | NR | 11.7 (IQR 63.3-186.6) | NR | 515.0 (IQR 365.0-755.0) | |
|  |  | 15 |  | 12.9 (IQR 11.3-26.0) |  | 0.5 (IQR 0.4-1.3) |  | 84.9 (IQR 73.5-186.6) |  | 784 (IQR 484.0-1,216.0) | |
| 4 | Han 2020 | NR | NR | NR | NR | NR | NR | NR | NR | NR | |
| 5 | Zhang 2020 | 8 | NR | NR | NR | 0.8 (IQR 0.6-1.1) | NR | 34.2 (IQR 12.5-67.4) | NR | NR | |
|  |  | 13 |  |  |  | 0.9 (IQR 0.6-1.4) |  |  |  |  |  |
| 6 | Shi 2020 | Before symptom onset | NR | NR | 1·1 (SD 0·3) | NR | 6·9 (SD 5·4) | NR | NR | NR | |
|  |  | ≤7 |  |  | 1·0 (SD 0·3) | NR | 61·4 (SD 39·6) | NR |  |  |  |
|  |  | ≥7-14 |  |  | NR | 1·1 (SD 0·3) | NR | 71·3 (SD 39·8) |  |  |  |
|  |  | ≥14-21 |  |  | NR | 1·1 (SD 0·3) | NR | 49·8 (SD 42·4) |  |  |  |
| 7 | Li 2020 | NR | NR | NR | NR | NR | NR | NR | NR | NR | |
| 8 | Huang 2020 | 7 | 5·0 (IQR 3·3–8·9) | NR | 0·8 (IQR 0·6–1·1) | NR | NR | NR | 286·0 (IQR 242·0–408·0) | NR | |
| 9 | Pan 2020 | 2 | 3.1 (SD 0.8) | NR | 1.4 (SD 0.5) | NR | 17.2 (SD 20.0) | NR | 242 (SD 73) | NR | |
| 10 | Chen 2020 | 4 | NR | NR | 1.12 (IQR 0.79-1.49) | NR | 12 (IQR 4.4-29.4) | NR | 229 (IQR 195-291) | NR | |
| 11 | Bernheim 2020 | NR | NR | NR | NR | NR | NR | NR | NR | NR | |
| 12 | Xiong 2020 | NR | NR | NR | NR | NR | NR | NR | NR | NR | |
| 13 | Wang 2020 | 7 | 3.0 (IQR 2.0-4.9) | NR | 0.8 (IQR 0.6-1.1) | NR | NR | NR | 261 (IQR 182-403) | NR | |
| 14 | Liu 2020 | NR | NR | NR | NR | NR | NR | NR | NR | NR | |
| 15 | Wang 2020 | NR | NR | NR | NR | NR | NR | NR | NR | NR | |
| 16 | Yuan 2020 | NR | NR | NR | NR | NR | NR | NR | NR | NR | |
| 17 | Xu 2020 | 2 | 2.9 (IQR 2.3-3.7) | NR | 1 (IQR 0.9-1.5) | NR | NR | NR | 194.5 (IQR 166.3-213.8) | NR | |
|  |  | 6.5 | 2.8 (IQR 1.7-3.9) |  | 1.0 (IQR 0.7-1.4) |  |  |  | 233.5 (IQR 198.0-312.3) |  |  |
| 18 | Salehi 2020 | NR | NR | NR | NR | NR | NR | NR | NR | NR | |
| 19 | Zhu 2020 | 5 | 3.7 (SD 1.9) | NR | 1.1 (SD 0.6) | NR | 20.7 (SD 24.0) | NR | 246.5 (SD 82.1) | NR | |
| 20 | Zhou 2020 | <4 | 0.71 (SD 0.13) | NR | 1.04  (SD 0.49) | NR | 15.98 (IQR 1.78–55.98) | NR | NR | NR | |
|  |  | 5-7 | 0.74 (SD 0.11) |  | 0.97 (SD 0.46) |  | 35.62 (IQR 5.63–63.04) |  |  |  |  |
| 21 | Song 2020 | NR | NR | NR | NR | NR | NR | NR | NR | NR | |
| 22 | Liu 2020 | NR | NR | NR | NR | NR | NR | NR | NR | NR | |
| 23 | Wang 2020 | 6 | 2.35 (IQR 1.62-3.67) | NR | 1.15 (IQR 0.82-1.46) | NR | 13.20 (IQR 6.78-49.00) | NR | 224.00 (IQR 183.00-291.00) | NR | |
| 24 | Liu 2020 | NR | NR | NR | NR | NR | NR | NR | NR | NR | |
| 25 | Li 2020 | NR | NR | NR | NR | NR | NR | NR | NR | NR | |
| 26 | Wu 2020 | 7 | 3.74 (IQR 2.67–5.20) | NR | 1.15 (IQR 0.76–1.40) | NR | 12.39 (IQR 2.71–50.61) | NR | NR | NR | |
| 27 | Liu 2020 | 8.58 | NR | 4.27 (SD 2.26),  3.36 (IQR 2.91-5.37) | NR | NR | NR | 41.1 (SD 26.4),  37.1 (IQR 24.6-53.1) | NR | 605 (SD 244), 576 (IQR 487-671) | |
| 28 | Wang 2020 | NR | NR | NR | NR | NR | NR | NR | NR | NR | |
| 29 | Fang 2020 | NR | NR | NR | NR | NR | NR | NR | NR | NR | |
| 30 | Bai 2020 | NR | NR | NR | NR | NR | NR | NR | NR | NR | |
| 31 | Liu 2020 | NR | NR | NR | NR | NR | NR | NR | NR | NR | |
| 32 | Yang 2020 | 6.85 | 2.60 (SD 2.03) | NR | 1.21 (SD 0.68) | NR | 7.25 (SD 23) | NR | 210 (SD 94.5) | NR | |
| 33 | Yang 2020 | 9 (survivors) | NR | NR | NR | 0.74 (SD 0.40) | NR | NR | NR | NR | |
|  |  | 11 (non-survivors) |  |  |  | 0.62 (SD 0.37) |  |  |  |  |  |
| 34 | Zhao 2020 | 5 | NR | NR | 0.97 (IQR 0.30-2.03) | NR | 26.47 (IQR 10-127.1) | NR | 256.94 (IQR 150-750) | NR | |
| 35 | Zhou 2020 | NR | NR | NR | NR | NR | NR | NR | NR | NR | |
