# Supplementary material for: Temporal changes of haematological and radiological findings of the COVID-19 infection—a review of literature
Source: BMC Pulm Med. 2021 Jan 22;21:37. doi: 10.1186/s12890-020-01389-z (PMC7820529; doi:10.1186/s12890-020-01389-z)
Supplement: Supplementary file 4 — Additional file 4. Comparison of blood and radiology findings between severe and non-severe patients. NR = not reported. [file 12890_2020_1389_MOESM4_ESM.docx]

*Additional file 4. Comparison of blood and radiology findings between severe and non-severe patients. NR = not reported.*

| **S/N** | **Code** | **Brief cohort grouping** | **Blood Results of Patients Stratified by Outcomes** | **Radiology Results of Patients Stratified by Outcomes** |
| --- | --- | --- | --- | --- |
| 3 | Zhang 2020 | Severity was established based on respiratory functions on admission with one of the below criteria: respiratory frequency ≥ 30/min, oxygen saturation ≤ 93% at rest, and oxygenation index ≤ 300 mm Hg | - Lower lymphocyte percentage (median, 0.7 vs 0.8, P = .048) - Noticeably, the percentages, but not absolute counts of lymphocytes, were lower in severe patients when compared to non-severe patients. This may be due to the increased total numbers of leukocytes in severe patients. - Higher median values of CRP (47.6 vs 28.7, P < .001) - may represent more prominent inflammation in severe patients. | NR |
| 5 | Huang 2020 | 13/42 patients (32%) were admitted to the ICU because they required high-flow nasal cannula or higher-level oxygen support measures to correct hypoxaemia | Neutrophil count: (p value 0·00069)   - ICU patients: 10·6 (5·0–11·8) - Non-ICU patients: 4·4 (2·0–6·1)   Lymphocyte count: (p value 0·0041)   - ICU patients: 0·4 (0·2–0·8) - Non-ICU patients: 1·0 (0·7–1·1)   LDH: (p value 0·0044)   - ICU patients: 400·0 (323·0–578·0) - Non-ICU patients: 281·0 (233·0–357·0) | - ICU patients: Bilateral multiple lobular and subsegmental areas of consolidation - 13/13 (100%) bilateral involvement - Non-ICU patients: Bilateral ground-glass opacity and subsegmental areas of consolidation - 27/28 (96%) bilateral involvement |
| 7 | Chen 2020 | ICU (ARDS) vs Non-ICU | In univariate analysis, high levels of lymphocyte cell count, CRP, lactate dehydrogenase (LDH) were all associated with the development of ARDS | NR |
| 9 | Xiong 2020 | Patients were not clearly stratified | CRP and LDH showed significant positive correlation with the severity of pneumonia quantified on initial CT. | As the disease progressed, severe cases had more consolidation and air bronchograms in the relevant lobes. The diffuse lesions, shown as “white lungs” were seen in the most severely affected patients. |
| 10 | Wang 2020 | ICU vs Non-ICU  Survivors vs Non-survivors | - ICU vs Non-ICU: Higher neutrophil counts in ICU patients - Survivors vs Non-survivors: More severe lymphopenia over time | NR |
| 13 | Yuan 2020 | Survival vs Mortality | NR | The median CT score of mortality group was higher compared to survival group (30 (IQR 7–13) vs 12 (IQR 11–43), P = 0.021), with more frequency of consolidation (40% vs 6%, P = 0.047) and air bronchogram (60% vs 12%, P = 0.025) |
| 18 | Wang 2020 | SpO2 <90 vs >90 | - Patients of the SpO2<90% group showed more frequency of lymphopenia than those of the SpO2≥90% group + lower lymph count in SPO2<90 (0.61) vs SPO2>90 (1.19) - SpO2<90% group has a higher neutrophil count (5.24) than SPo2>90% group (2.16) + more frequency - The proportion and extent of the increase of lactate dehydrogenase, c reactive protein, and erythrocyte sedimentation rate are more prominent in the SpO2<90% group | The opacity area of the ground glass is larger and the consolidation degree is more serious in the SpO2<90% group. |
| 19 | Li 2020 |  |  | - Compared with the severe-critical type, the common type had a lower incidence of right upper lobe and middle lobe involvement, and also a lower incidence of right lower lobe, left lower lobe, and left upper lobe involvement - Common type and severe-critical type can both involve 5 lobes, but severe-critical type had a higher incidence than common type |
| 21 | Liu 2020 | Murray score | - Lymphocyte counts were negatively correlated with Murray scores - LDH and CRP positively correlated with Murray score |  |
| 25 | Yang 2020 | Survivors vs Non-survivors (but all were critically ill) | - No significant difference between the 2 groups (between survivors vs non-survivors) - Lymphocytopenia occurred in more than 80% of critically ill patients in our cohort. Lymphocytopenia is a prominent feature of critically ill patients with SARS-CoV infection because targeted invasion by SARS-CoV viral particles damages the cytoplasmic component of the lymphocyte and causes its destruction. - This is compared to a previous study, mainly in non-critical patients infected with SARS-CoV-2, 35% of patients had only mild lymphocytopenia |  |
