# Supplementary material for: Temporal changes of haematological and radiological findings of the COVID-19 infection—a review of literature
Source: BMC Pulm Med. 2021 Jan 22;21:37. doi: 10.1186/s12890-020-01389-z (PMC7820529; doi:10.1186/s12890-020-01389-z)
Supplement: Supplementary file 5 — Additional file 5. Temporal changes of blood and radiology results stratiied by patients' outcocmes. NR = not reported. [file 12890_2020_1389_MOESM5_ESM.docx]

*Additional file 5. Temporal changes of blood and radiology results stratified by patients’ outcomes. NR = not reported*

| **S/N** |  | **Brief cohort grouping** | **Blood Results of Patients Stratified by Outcomes** | **Radiology Results of Patients Stratified by Outcomes** |
| --- | --- | --- | --- | --- |
| 1 | Zhang 2020 | All patients died in this study cohort | On the admission:   - Lymphopenia (89.2%) - Neutrophilia (74.3%) - Increased C-reactive protein level (100%) - High lactate dehydrogenase (93.2%)   In the last 24 hours of the death:   - Lymphopenia (73.7%) - Neutrophilia (100%) - Increased C-reactive protein level (100%) - High lactate dehydrogenase (100%) | NR |
| 3 | Zhang 2020 | Severity was established based on respiratory functions on admission with one of the below criteria: respiratory frequency ≥ 30/min, oxygen saturation ≤ 93% at rest, and oxygenation index ≤ 300 mm Hg | On admission, lymphocyte percentage:   - Non-severe patients: 20.0 (12.5-28.4) - Severe patients: 12.7 (7.7-22.0)   Subsequent test, lymphocyte percentage:   - Non-severe patients: 22.1 (13.5-29.5) [Median days from previous test: 5 (4.0-10.0)] - Severe patients: 10.3 (4.3-16.6) [Median days from previous test: 5 (3-7)] | NR |
| 10 | Wang 2020 | Survivors vs Non-survivors | - During hospitalization, most patients had marked lymphopenia, and non-survivors developed more severe lymphopenia over time - White blood cell counts and neutrophil counts were higher in non-survivors than those in survivors | NR |
| 13 | Yuan 2020 | Survival vs Mortality | NR | Re-examination:   - Survival: The morphology of the lesions, the location, extent, and distribution of involvement of each abnormality were not significantly changed compared to those on admission - Mortality: CT scores progressed rapidly in a short time (12 (IQR5–24.5) vs 20 (IQR 15–46), P = 0.042), with more lung zones being involved |
