# Supplementary material for: Temporal changes of haematological and radiological findings of the COVID-19 infection—a review of literature
Source: BMC Pulm Med. 2021 Jan 22;21:37. doi: 10.1186/s12890-020-01389-z (PMC7820529; doi:10.1186/s12890-020-01389-z)
Supplement: Supplementary file 6 — Additional file 6. Chest CT imaging findings for early phase. NR = not reported. [file 12890_2020_1389_MOESM6_ESM.docx]

*Additional file 6. Chest CT imaging findings for early phase. NR = not reported.*

|  |  | **Lung changes** | | | | **Lesion distribution** | | | | | | | |
| --- | --- | --- | --- | --- | --- | --- | --- | --- | --- | --- | --- | --- | --- |
| **S/N** |  | **GGO** | **Consolidation** | **GGO + Consolidation** | **Crazy paving pattern** | **Bilateral lungs** | **Unilateral lung** | **No of lobes: 1** | **No of lobes: 2 or 3** | **No of lobes: 4 or 5** | **Peripheral** | **Central** | **Peripheral + Central** |
| 2 | Han 2020 | 65/108 (60.2%) | 6/108  (5.56%) | 44/108 (40.7%) | 43/108 (39.8%) | NR | NR | 38/108 (35.2%) | 24/108 (22.2%) | 46/108 (42.6%) | 97/108 (89.8%) | 2/108 (1.85%) | 9/108 (8.33%) |
| 4 | Shi 2020 | 31/36 (86.1%) | 2/36  (5.56%) | NR | 2/36  (5.56%) | 25/36 (69.4%) | 11/36 (30.6%) | NR | NR | NR | 24/36 (66.7%) | 8/36 (22.2%) | 4/36 (11.1%) |
| 5 | Huang 2020 | NR | 13/41  (31.7%) | 28/41  (68.3%) | NR | 40/41 (97.6%) | NR | NR | NR | NR | NR | NR | NR |
| 6 | Pan 2020 | 32/41 (78.0%) | 18/41  (43.9%) | NR | 15/41  (36.65%) | NR | NR | 14/41 (34.1%) | NR | NR | NR | NR | NR |
| 7 | Chen 2020 | NR | NR | NR | NR | 203/249 (81.5%) | 39/249 (15.7%) | NR | NR | NR | NR | NR | NR |
| 8 | Bernheim 2020 | 45/69 (65.2%) | 24/69  (34.8%) | NR | 1/69  (1.45%) | 35/69 (50.7%) | NR | 10/69 (14.5%) | 14/69 (20.3%) | 22/69 (31.9%) | 29/69 (42.0%) | 0/69 (0%) | NR |
| 9 | Xiong 2020 | NR | 23/42  (54.8%) | NR | NR | NR | NR | 10/42 (23.8%) | 4/42  (9.52%) | 28/42 (66.7%) | 12/42 (28.6%) | 5/42 (11.9%) | 25/42 (59.5%) |
| 10 | Wang 2020 | 138/138 (100%) | NR | NR | NR | 138/138 (100%) | 0/138 (0%) | NR | NR | NR | NR | NR | NR |
| 11 | Liu 2020 | 43/55 (78.2%) | 25/55  (45.5%) | 33/55  (60%) | 20/55  (36.4%) | 37/55 (67.3%) | 15/55 (27.3%) | 11/55 (20%) | 16/55 (29.1%) | 25/55 (45.5%) | 54/55 (98.2%) | 1/55 (1.82%) | NR |
| 12 | Wang 2020 | 51/89 (57.3%) | 20/89  (22.5%) | NR | 1/79  (1.27%) | 39/74 (52.7%) | 18/66 (27.3%) | NR | NR | NR | NR | NR | NR |
| 15 | Zhu 2020 | 4/32 (12.5%) | 2/32  (6.25%) | NR | 1/32  (3.13%) | 29/32 (90.6%) | NR | NR | NR | NR | NR | NR | NR |
| 16 | Zhou 2020 | 38/62 (61.3%) | 1/62  (1.61%) | 22/62  (35.5%) | 16/62  (25.8%) | NR | NR | NR | NR | NR | 21/62 (33.9%) | 2/62 (3.22%) | 39/62 (62.9%) |
| 17 | Song 2020 | 39/51 (76.5%) | 28/51  (54.9%) | 30/51  (58.8%) | 38/51  (74.5%) | 44/51 (86.3%) | 7/51 (13.7%) | 4/51 (7.84%) | 14/51 (27.5%) | 32/51 (62.7%) | 44/51 (86.3%) | 5/51 (9.80%) | 1/51 (1.96%) |
| 19 | Li 2020 | 45/78 (57.7%) | 12/78  (15.4%) | NR | NR | 45/78 (57.7%) | NR | 8/78 (10.3%) | 11/78 (14.1%) | 35/78 (44.9%) | 49/56 (87.5%) | 23/78 (29.5%) | NR |
| 20 | Wu 2020 | 73/80 (91.3%) | 50/80  (62.5%) | NR | 23/80  (28.8%) | NR | NR | NR | NR | NR | NR | NR | NR |
| 22 | Bai 2020 | 200/219 (91.3%) | 150/219 (68.5%) | NR | 11/219 (5.02%) | 165/219 (75.3%) | 41/219 (18.7%) | NR | NR | NR | 176/219 (80.4%) | 3/219 (1.37%) | 31/219 (14.2%) |
| 23 | Liu 2020 | 4/10 (40%) | 5/10  (50%) | NR | NR | NR | NR | NR | NR | NR | NR | NR | NR |
| 27 | Zhou 2020 | 19/40 (47.5%) | 15/40  (37.5%) | NR | 20/40  (50%) | NR | NR | NR | NR | NR | NR | NR | NR |
