# Supplementary material for: Temporal changes of haematological and radiological findings of the COVID-19 infection—a review of literature
Source: BMC Pulm Med. 2021 Jan 22;21:37. doi: 10.1186/s12890-020-01389-z (PMC7820529; doi:10.1186/s12890-020-01389-z)
Supplement: Supplementary file 7 — Additional file 7. Chest CT imaging findings for late phase. NR = not reported. [file 12890_2020_1389_MOESM7_ESM.docx]

*Additional file 7. Chest CT imaging findings for late phase. NR = not reported.*

| Author | **Lung changes** | | | | **Lesion distribution** | | | | | | | |
| --- | --- | --- | --- | --- | --- | --- | --- | --- | --- | --- | --- | --- |
|  | **GGO** | **Consolidation** | **GGO + Consolidation** | **Crazy paving pattern** | **Bilateral lungs** | **Unilateral lung** | **No of lobes: 1** | **No of lobes: 2 or 3** | **No of lobes: 4 or 5** | **Peripheral** | **Central** | **Peripheral + Central** |
| Zhang 2020 | NR | NR | NR | NR | 121/135 (89.6%) | 13/135 (9.63%) | NR | NR | NR | NR | NR | NR |
| Shi 2020 | 22/45 (48.9%) | 12/45  (26.7%) | NR | 6/45  (13.3%) | 39/45 (86.7%) | 6/45 (13.3%) | NR | NR | NR | 20/45 (44.4%) | 2/45 (4.44%) | 23/45 (51.1%) |
| Pan 2020 | 28/41 (68.3%) | 34/41  (82.9%) | NR | 4/41  (9.76%) | NR | NR | 7/41 (17.1%) | NR | NR | NR | NR | NR |
| Bernheim 2020 | 22/25 (88%) | 15/25  (60%) | NR | 5/25  (20%) | 22/25 (88%) | NR | 1/25 (4%) | 4/25  (16%) | 19/25  (76%) | 18/25 (72%) | 0/25 (0%) | NR |
| Xiong 2020 | NR | 34/42  (81.0%) | NR | NR | NR | NR | 3/42 (7.14%) | 2/42  (4.76%) | 37/42  (88.1%) | 7/42 (16.7%) | 1/42 (2.38%) | 34/42 (81.0%) |
| Wang 2020 | 68/132 (51.5%) | 20/85  (23.5%) | NR | 42/132 (31.8%) | 218/229 (95.2%) | NR | NR | NR | NR | NR | NR | NR |
| Yuan 2020 | 18/27 (66.7%) | 5/27  (18.5%) | 8/27  (29.6%) | NR | 23/27 (85.2%) | 4/27 (14.8%) | NR | NR | NR | 7/27 (25.9%) | 0/27 (0%) | 20/27 (74.1%) |
| Zhou 2020 | 6/22 (27.3%) | 6/22  (27.3%) | NR | 19/22  (86.4%) | NR | NR | NR | NR | NR | NR | NR | NR |
